# The analysis of GM (1, 1) grey model to predict the health resource allocation in Jilin Province, China: An observational study

**DOI:** 10.1097/MD.0000000000039298

**Published:** 2024-08-23

**Authors:** Wanxu Pu, Xitao Yue, Qi Xiong, Kaikai Jia, Yuanjun Zou

**Affiliations:** a Changchun University of Chinese Medicine, Changchun, Jilin Province, China.

**Keywords:** GM (1, 1) grey model, health human resources, health physical resources, prediction analysis

## Abstract

To predict the development of health resource allocation in Jilin Province during the 14th 5-Year Plan period, and to provide a scientific basis for promoting the improvement of its service capacity. The data of the health resource from 2015 to 2022 were obtained from the Jilin Statistical Yearbook, and the number of medical institutions, medical beds, health technicians, licensed (assistant) physicians, registered nurses and pharmacists were selected as evaluation indicators, and the grey prediction model constructed by Python was used to predict the development from 2023 to 2025. In the 14th 5-Year Plan period, the health resource in Jilin Province showed an increasing trend, and it is predicted that in 2025, the number of medical institutions, medical beds, health technicians, licensed (assistant) physicians, registered nurses, and pharmacists in Jilin Province will reach 28,999, 196,328, 262,219, 101,273, 129,586, and 9469, respectively. Except that the pharmacist team failed to meet the planning objectives of the 14th 5-Year Plan, the remaining health resources could meet the planning requirements. The allocation level of health resources in Jilin Province has been continuously improved, but it still faces the problems that the allocation of medical beds needs to be optimized, the doctor-nurse ratio needs to be improved, the reserve of registered nurses is insufficient, there is a gap in the pharmacist team, and the development of pharmacy services is slow.

## 1. Introduction

The allocation of health resources is recognized as the basis for the sustainable development of health undertakings,^[1]^ and the rationality of their allocation affects residents’ health levels. The scientific assessment of the supply–demand matching of health resources is of great theoretical and practical significance to reasonably allocate and lay out health resources, enhance their service capacity, thus raising the standard of health care services and lengthening citizens’ healthy lives.^[2]^ At present, how to evaluate and optimize the input structure of health resources and improve the efficiency of health resource utilization is one of the important topics in the research of China’s health service sustainability.^[3]^

Jilin Province, the geometric center of northeast Asia, lies in the central part of northeast China, neighboring the far east of Russia and Korean peninsula to its east and southeast and with Japan on the other side of the Sea of Japan.^[4]^ Under the background of Northeast Revitalization Strategy, promoting the construction of healthy Jilin is one of the key tasks. At this stage, more research focuses on the epidemiological investigation of Jilin Province,^[5–9]^ and few studies have been conducted on its health resource allocation. Therefore, in the context of attaching great importance to the service ability of health resources, it is extremely urgent to analyze the allocation of health resources and development projections in Jilin Province.

In recent years, the grey model first-order one variable GM (1, 1) has been widely used in the field of medicine and health.^[10,11]^ It is a time series forecasting model,^[12]^ which has the characteristics of small amount of original sample data, high short-term prediction accuracy and good effect.^[13,14]^

In this study, the GM (1, 1) model was proposed to make prediction of the future trend of health resources in Jilin Province, and provide reference for the government in policy making. The data comes from the statistical data published by the local government, and the mode is constructed by Python. In addition, this study further proves that GM (1, 1) is suitable for the prediction of health resource demand.

The remaining sections of the paper are organized as follows: Section 2 gives the literature review and the reasons for using GM (1, 1). Section 3 covers data presentation and method introduction. Section 4 describes the development of the research object. Section 5 discusses the research findings and gives reasonable policy guidance. Section 6 concludes the study.

## 2. Literature review

The “Healthy China 2030” strategy is an important measure for China to actively participate in global health governance and fulfill its commitment to the United Nations “2030 Agenda for Sustainable Development.”^[15]^ The strategy directs that the health needs of the people be met, basic medical and health services be made available to all.^[16]^ Since the new medical reform in China, although the unfairness of medical and health resources has gradually decreased, the phenomenon of uneven distribution of resources still exists.^[17]^ For example, there are huge differences in the allocation of healthcare resources between urban and rural areas.^[18]^ How to maximize the accessibility of health services in the case of limited medical resources? How to allocate health resources more reasonably?

Due to the limited resources, hospital and health system managers are faced with the problem of how to balance resources between different services and professions. Many scholars have conducted in-depth research on this issue. Ordu et al^[19]^ developed a whole hospital-level decision support system to assess and respond to the needs of local populations to help make efficient and effective use of scarce medical resources. Aiming at the daily functioning of a hospital, the team proposed a hybrid forecasting–simulation–optimization model to improve the resource allocation in a hospital setting.^[20]^ Kirli Akin et al^[21]^ developed a novel simulation-based 2-stage optimization approach to produce a balanced scheduling between the nurses. Latruwe et al^[22]^ developed a hybrid model to provide a strategic planning for bed capacity in a hospital.

The rational allocation of healthcare resources is essential to ensure that all patients can obtain the necessary medical services. Many methods and indicators are used to study the equity of health resource allocation, such as Concentration Index,^[23]^ Lorenz Curve,^[1]^ Gini Coefficient,^[24]^ and Theil Index.^[25]^ Each method and index have its own advantages and disadvantages, and the applicable conditions are not the same.^[26]^ However, these methods are mainly used to describe and analyze the distribution characteristics of data, especially in the study of income distribution and health inequality. The description of the distribution of existing resources does not directly provide a prediction of future trends. Therefore, they are used more to assess and explain the status quo than to predict future changes.

In terms of forecasting techniques, there are ARIMA, exponential smoothing and linear regression methods. Each method has its own applicable conditions. ARIMA is suitable for the case of large samples, if the sample size of the data is small, this will limit the use of the model.^[27]^ Although exponential smoothing has certain flexibility in processing time series data, it usually needs to adjust the volatility of the data, and has the best effect in the absence of obvious trends and seasonality.^[28]^ Unfortunately, there is a certain development trend in health resources. Linear regression requires a model based on data that must satisfy specific statistical assumptions, and it is not suitable for cases where there is a certain degree of uncertainty and incompleteness of the data.^[29]^

In this study, we are faced with a typical small sample, incomplete information time series prediction problem. Considering the particularity of the data, we choose the GM (1, 1) model as the prediction tool.

## 3. Materials and methods

### 3.1. Data sources

The data of health resources were taken from the Jilin Statistical Yearbook in 2016 to 2023 (2023 is the latest release), which covered permanent population, health physical resources and health human resources in Jilin Province. The medical institutions and beds represent health physical resources, and health technicians, licensed (assistant) physicians, registered nurses and pharmacists represent health human resources. This is a longitudinal study to analyze the change of health resource allocation of Jilin Province from 2015 to 2022 and to predict the development of the 14th 5-Year Plan period.

### 3.2. Indicators

According to a previous related study,^[30]^ health physical resources and health human resources are important indicators of health services, the number of medical institutions, medical beds, health technicians, licensed (assistant) physicians, registered nurses, and pharmacists from 2015 to 2022 were selected as evaluation indicators.

### 3.3. Research methods

**Figure 1.**
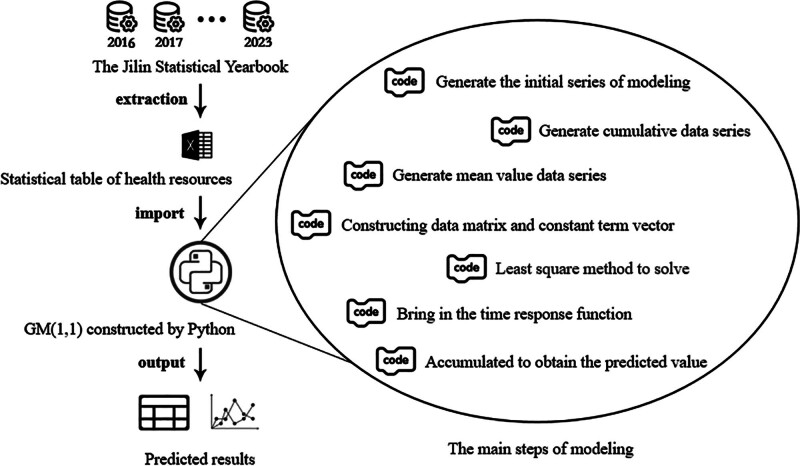
The method processing of this study. This figure fully shows the process of work in this paper. Excel is used to collect and sort out the data set published by the government over the years, and Python programming is used to realize the GM (1, 1) prediction model. The model will read Excel and output the prediction results. The right picture introduces the key steps of the model from top to bottom.

Descriptive analysis was used to evaluate the change of health resource allocation of Jilin Province. Grey prediction model GM (1,1) was used to predict the development of health resources of Jilin Province during the 14th 5-Year Plan period. The process of this study is shown in Figure 1. The main process of modeling is as follows:

(1) Generate the initial series of modeling:


$$\begin{array}{l} X^{\left(0\right)}=\left[x^{\left(0\right)}\left(1\right),\;x^{\left(0\right)}\left(2\right),\ldots ,x^{\left(0\right)}\left(n\right)\right]\; \end{array}$$

(2) Generate cumulative data series:


$$\begin{array}{l} X^{\left(1\right)}=\left[x^{\left(1\right)}\left(1\right),\;x^{\left(1\right)}\left(2\right),\ldots ,x^{\left(1\right)}\left(n\right)\right] \end{array}$$

(3) Generate mean value data series:


$$\begin{array}{l} \;\;\;\;Z^{\left(1\right)}=\left[z^{\left(1\right)}\left(2\right),\;z^{\left(1\right)}\left(3\right),\ldots ,z^{\left(1\right)}\left(n\right)\right] \end{array}$$

(4) Constructing data matrix (*B*) and constant term vector (*Y*)


$$B=\left(\begin{array}{c} \begin{array}{cc} -z^{\left(1\right)}\left(2\right) & 1\\ -z^{\left(1\right)}\left(3\right) & 1 \end{array}\\ \begin{array}{cc} \vdots & \vdots \\ -z^{\left(1\right)}\left(n\right) & 1 \end{array} \end{array}\right),\;Y=\left(\begin{array}{c} x^{\left(0\right)}\left(2\right)\\ x^{\left(0\right)}\left(3\right)\\ \vdots \\ x^{\left(0\right)}\left(n\right) \end{array}\right)$$

(5) Least square method to solve:


$$\begin{array}{l} \hat{a}={\left(B^{T}B\right)}^{-1}B^{T}Y \end{array}$$

(6) Bring in the time response function:


$$\begin{array}{l} {\hat{x}}^{\left(1\right)}\left(k+1\right)=\left(x^{\left(0\right)}\left(1\right)-\frac{b}{a}\right)e^{-ak}+\frac{b}{a} \end{array}$$

(7) Accumulated to obtain the predicted value:


$$\begin{array}{l} {\hat{x}}^{\left(0\right)}\left(k+1\right)={\hat{x}}^{\left(1\right)}\left(k+1\right)-{\hat{x}}^{\left(1\right)}\left(k\right) \end{array}$$

### 3.4. Accuracy tests on the model

A scientific and reasonable GM (1, 1) model needs to pass 2 model accuracy tests, namely small error probability (*P*) and posterior error ration (*C*), before it is applied to actual prediction.^[31]^ The accuracy grade confirmation of the model is shown in Table 1.

**Table 1:** Judgment standard of prediction accuracy for GM (1, 1).

Prediction accuracy grades	Small error probability (*P*)	Posterior error ration (*C*)
1. Excellent	*P* ≥ 0.95	*C* ≤ 0.35
2. Qualified	0.95 > *P* ≥ 0.80	0.35 < *C* ≤ 0.50
3. Barely qualified	0.80 > *P* ≥ 0.70	0.50 < *C* ≤ 0.65
4. Unqualified	0.70 > *P*	0.65 < *C*

The table gives 4 grades of GM (1, 1), which are tested by the small error probability (*P*) and posterior error ration (*C*) of the 2 evaluation model standard.

## 4. Results

### 4.1. Status of health resource allocation in Jilin Province

The number of health resource allocations in Jilin Province from 2015 to 2022 shrinks in individual years, but generally shows an upward trend. Compared with 2015, in 2022, the number of medical institutions in Jilin Province increased by 4412, the number of beds increased by 32,298, the number of health technicians increased by 58,876, the number of licensed (assistant) physicians increased by 19,382, the number of registered nurses increased by 37,897, and the number of pharmacists increased by 828 (Table 2).

**Table 2:** Basic information of health resources.

Year	Medical institutions	Medical beds	Health technicians	Licensed (assistant) physicians	Registered nurses	Pharmacists
2015	20,619	144,695	159,103	67,279	60,742	7903
2016	20,828	151,155	166,527	69,613	65,736	7931
2017	20,827	153,625	168,080	70,564	67,142	7900
2018	22,648	166,743	183,327	76,910	76,140	8139
2019	22,178	170,582	188,257	78,741	78,870	8078
2020	25,626	173,143	212,140	85,123	95,392	8717
2021	25,345	176,306	217,215	87,315	97,803	8894
2022	25,031	176,993	217,979	86,661	98,639	8731

The table shows the results of data extracted and collated from the Jilin Statistical Yearbook.

### 4.2. Grey model construction and testing

In this paper, the GM (1, 1) model was constructed with the health resources of Jilin Province as the initial sequence of modeling in 2015 to 2022, and the model was tested for prediction accuracy. The results show that the *C* value of medical institutions is 0.1535 and the *P* value is 0.875, which means that the model accuracy reaches the qualified level. The *C* value of the rest of the indexes are all <0.35, and the *P* value is more than 0.95, indicating that the model fitting effect is good. The development coefficients of all models 𝑎 ≥ −0.3 and the relative error rates are within the acceptable range,^[32]^ indicating that the constructed models have high prediction accuracy and can be used for extrapolation prediction (Tables 3 and 4).

**Table 3:** Prediction model and test results of health resources.

Predictive Indicators	Parameter value	*C*	*P*	Accuracy level
Medical institutions	𝑎 = −0.0376	0.1535	0.875	Level 2
	*b* = 19515.8506			
Medical beds	𝑎 = −0.0273	0.0711	1.000	Level 1
	*b* = 147551.9272			
Health technicians	𝑎 = −0.0517	0.0497	1.000	Level 1
	*b* = 152234.9888			
Licensed (assistant) physicians	𝑎 = −0.0414	0.0522	1.000	Level 1
	*b* = 65552.8446			
Registered nurses	𝑎 = −0.0766	0.0562	1.000	Level 1
	*b* = 57921.8695			
Pharmacists	𝑎 = −0.0213	0.1531	1.000	Level 1
	*b* = 7566.6949			

The table gives the model parameter values of the 6 indicators and the test results of the model prediction accuracy.

**Table 4:** Prediction results of health resources from 2015 to 2025.

Year	Medical institutions		Medical beds		Health technicians		Licensed (assistant) physicians		Registered nurses		Pharmacists	
	Predictive value	Relative error (%)	Predictive value	Relative error (%)	Predictive value	Relative error (%)	Predictive value	Relative error (%)	Predictive value	Relative error (%)	Predictive value	Relative error (%)
2015	20,619	0.000	144,695	0.000	159,103	0.000	67,279	0.000	60,742	0.000	7903	0.000
2016	20,677	0.726	153,585	1.608	164,678	1.110	69,772	0.229	65,034	1.068	7818	1.426
2017	21,469	3.081	157,833	2.739	173,413	3.173	72,721	3.057	70,212	4.572	7986	1.090
2018	22,291	1.576	162,198	2.726	182,612	0.390	75,795	1.450	75,802	0.444	8158	0.233
2019	23,145	4.359	166,684	2.285	192,299	2.147	78,999	0.327	81,836	3.761	8334	3.163
2020	24,031	6.224	171,293	1.068	202,500	4.544	82,338	3.272	88,352	7.380	8513	2.342
2021	24,951	1.553	176,031	0.156	213,242	1.829	85,818	1.715	95,386	2.471	8696	2.226
2022	25,907	3.500	180,899	2.207	224,554	3.016	89,445	3.213	102,980	4.401	8883	1.743
2023	26,899	–	185,902	–	236,466	–	93,226	–	111,178	–	9074	—
2024	27,929	–	191,044	–	249,010	–	97,166	–	120,030	–	9270	—
2025	28,999	–	196,328	–	262,219	–	101,273	–	129,586	–	9469	—

The table gives the prediction results of the 6 indicators, and compares the real values from 2015 to 2022 to calculate the relative error rate.

### 4.3. Projections of health resources in Jilin Province

According to the model prediction results, medical resources in Jilin Province will continue to develop and show growth from 2023 to 2025 (Table 4). Combining with the prediction data of the local permanent population can bring more valuable assessment. It is expected that by 2025, the permanent population of Jilin Province will reach 22,343,500 people, and compared with the 14th 5-Year Plan, it is found that, except for the pharmacist indicator, all the other indicators exceed the development goal of the plan (Table 5).

**Table 5:** Expected completion of the 14th 5-Year Plan development goals.

Indicators	Target 2025	Projected result 2025
Number of medical beds per thousand population	7.50	8.78
Total number of licensed (assistant) physicians	85,100	101,273
Number of licensed (assistant) physicians per thousand population	3.54	4.53
Total number of registered nurses	110,000	129,586
Number of registered nurses per thousand population	4.20	5.80
Doctor-nurse ratio	1:1.20	1:1.27
Total number of pharmacists	13,000	9469
Number of pharmacists per thousand population	0.54	0.42

The table shows the comparison between the predicted results of the 8 indicators and the requirements of regional development planning. It can be found that the pharmacist indicators fail to meet the requirements.

## 5. Discussion

From the perspective of the GM (1, 1) model, the development coefficient 𝑎 needs to be between −2 and 2 in order to be meaningful, and the models constructed in this paper all satisfy the interval requirement and the results of the model test show that the model fitting accuracy of medical institutions is qualified, and the model fitting accuracy of the rest of the indicators are all of level 1 (excellent). Therefore, the prediction accuracy of the 6 indicators is good, and the prediction results have a certain degree of accuracy, which can provide scientific reference for further optimizing the allocation of health resources and improving the medical service capacity in Jilin Province.

From the perspective of health physical resources, Jilin Province needs to strengthen the high-quality development of public hospitals and increase the optimal allocation of medical bed resources. The prediction results show that the number of medical institutions and beds in Jilin Province is on the rise from 2023 to 2025. Medical institutions mainly include hospitals, primary health care institutions, professional public health institutions and other health institutions in China. This study found that the changes in the number of medical institutions in Jilin Province are mainly concentrated in primary health care institutions, while the number of hospitals is slowing growth, and the number of professional public health institutions is gradually decreasing. The reasons for this phenomenon is closely related to the provincial government’s in-depth promotion of the construction of Healthy Jilin, regulating the resource structure measures. Through the model prediction, it can be seen that the medical beds in Jilin Province will exceed the 14th 5-Year Plan target, and the incremental beds will be guided by the planning policy,^[33]^ mainly invested in infectious, severe, tumor, mental, rehabilitation, pediatric. This study found that the number of hospital beds in Jilin Province accounted for about 85% of the total number of beds in the province’s medical institutions. Relevant literature shows that the bed resources in Jilin Province have not been fully utilized,^[34]^ such as poor allocation of severe beds. The reasons for this phenomenon may be related to the excessive concentration of bed resources in traditional departments, the lack of overall consideration of the characteristics of different departments by medical institutions, and the fact that public hospitals with long hospital construction time are often affected by historical distribution.^[35]^ Therefore, the first suggestion is to continue to promote the transformation of public hospitals from extension expansion to connotation improvement, break the inertia of hospital development path dependence on scale development, strengthen the dominant position of public hospitals in the medical service system, promote the expansion and sinking of high-quality medical resources, and improve the capacity of primary medical services. The second suggestion is to increase the optimization of the number of medical beds, improve the utilization efficiency of bed resources, and avoid the coexistence of shortage and idleness.

From the perspective of health human resources, the reserve of pharmacist team in Jilin Province is insufficient, and the ratio of doctors to nurses needs to be improved. It is necessary to fill the shortage of pharmacist talents and expand the nurse team. The prediction results show that the total amount of health human resources in Jilin Province will show a steady growth trend in 2023 to 2025, but the pharmacist team has not reached the development requirements of the 14th 5-Year Plan and there is a certain gap. The main reason is that the role of pharmacists as health care professionals is not deemed important by the medical institutions in China.^[36]^ At this stage, Chinese pharmacists are in a period of development and transformation, from focusing on the drug itself to focusing on the rational use of drugs, forming a patient-centered, out of the pharmacy, clinical-oriented pharmaceutical care concept.^[37]^ Pharmacists play a role in ensuring the safety, effectiveness, and economy of clinical medication.^[38]^ The development of the pharmacist workforce reflects trends in the demand for pharmacy services in healthcare organizations. It is suggested that there are some problems in the development of pharmaceutical industry in Jilin Province, and the understanding of the value of pharmacists in medical institutions is still insufficient. According to the model prediction, the number of registered nurses in Jilin Province will reach 129,568 in 2025, and the doctor–nurse ratio will reach 1:1.27, which is enough to complete the expected goal of the 14th 5-Year Plan of Jilin Province. However, there is still a big gap between the doctor–nurse ratio and the national standard of 1:2, which is a common problem in many provinces of China. The reason is related to the late start of nursing education in China, which leads to the great impact on the development of nursing human resources and the long-standing improper concepts of “heavy treatment, light nursing,” “heavy surgery, light rehabilitation.” At present, the nurses in Jilin Province generally have a good sense of professional identity,^[39]^ but there are also serious job burnout and low mental health.^[40]^ This is closely related to the high workload of nursing staff, shift system, doctor-patient contradiction and other reasons, which is easy to induce the problem of high turnover rate. Therefore, it is suggested that the first is to continuously enrich the pharmacist team and attach importance to the development and transformation of pharmacists in medical institutions to provide high-quality clinical pharmaceutical services. The second is to continue to increase the training of nursing talents to alleviate the shortage of nursing staff. The third is to focus on improving the job satisfaction of the nurse group to avoid the loss of nursing staff.

## 6. Conclusion

Our research shows that there is much room for future growth in the demand for health resources in Jilin Province, especially health human resources. Under the strategic background of “Healthy China 2030,” Jilin Province urgently needs to solve the problems of unreasonable bed allocation, doctor–nurse ratio to be further improved, insufficient reserve of registered nurses, shortage of pharmacists, and slow development of pharmaceutical services. In addition, it is noted that since the model mainly reflects the regularity of the data, it cannot fully reflect the impact of various non-regular social factors (such as policy, economy) on the prediction indicators, and the prediction value of the indicators has certain errors, which is also the deficiency of this paper. In future research, the model can be optimized or multiple prediction models can be considered to construct a combined model to further reduce the error.

## Author contributions

**Data curation:** Wanxu Pu.

**Methodology:** Wanxu Pu.

**Validation:** Wanxu Pu.

**Visualization:** Wanxu Pu.

**Writing – original draft:** Wanxu Pu.

**Project administration:** Xitao Yue.

**Investigation:** Qi Xiong.

**Conceptualization:** Kaikai Jia.

**Funding acquisition:** Yuanjun Zou.

**Writing – review & editing:** Yuanjun Zou.
